# Client-Seeking Environments, Safety Management, and Survival Strategies Among African male and gender-diverse refugee/migrant workers in Northern Italy: A Theory of Planned Behavior Mixed-Methods Study. BSGH026

**DOI:** 10.21203/rs.3.rs-9202242/v1

**Published:** 2026-03-24

**Authors:** Osman Wumpini Shamrock, Gamji Rabiu Abu-Ba’are, Samira Shirzaei Nichols, Marco Barracchia, George Rudolph Agbemedu, Charles Gagbe Nukunu, Adams Al-Mardhiyyah

**Affiliations:** 1. Behavioral, Sexual, and Global Health Lab, School of Nursing, University of Rochester Medical Center, Rochester, New York, United States; 2. Department of Public Health Sciences, University of Rochester Medical Center, Rochester, New York, United States; 3. Center for Interdisciplinary Research on AIDS, School of Public Health, Yale University, New Haven, Connecticut, United States; 4. Department of International Health, Care and Public Health Research Institute (CAPHRI) Maastricht University, Maastricht, The Netherlands; 5. Department of Computer Information Systems & Analytics, College of Business, University of Central Arkansas, Arkansas, United States; 6. University of Miami, School of Nursing and Health Sciences

**Keywords:** migrant sex workers, refugee populations, condom use, STI disclosure, digital sex work, peer networks, HIV risk, Theory of Planned Behavior, Italy

## Abstract

**Background:**

African male and gender-diverse refugee/migrant workers in Europe face intersecting structural vulnerabilities that shape HIV/STI risk. Client-seeking environments may influence sexual health behaviors, yet limited evidence examines how venue-based strategies affect condom use, STI disclosure, and risk negotiation.

**Methods:**

We conducted a sequential exploratory mixed-methods study among African male and gender-diverse refugee/migrant workers in Northern Italy. Qualitative data from 20 in-depth interviews and 2 focus group discussions informed a structured survey administered to 150 participants. Guided by the Theory of Planned Behavior, we examined associations between client-seeking venues and sexual health outcomes, including condom use, STI disclosure, awareness of clients’ STI status, and engagement in unprotected sex under financial pressure. Fisher’s Exact Test and two-proportion z-tests were used (p < 0.05).

**Results:**

Referral-based networks were the most common client-seeking strategy (62.7%), followed by bars/clubs (14.0%), street-based work (13.3%), online platforms (6.7%), and brothels (3.3%). Client-seeking venue was significantly associated with awareness of clients’ STI status (p = 0.003), condom use (p = 0.009), STI disclosure (p = 0.005), and acceptance of unprotected sex (p = 0.011). Online-based sex work was associated with the lowest condom use and STI disclosure, while brothel-based work showed higher reported condom use and STI awareness. Qualitative findings indicated that differences in peer support, negotiation dynamics, and perceived control across venues shaped these outcomes.

**Conclusions:**

Client-seeking environments are critical determinants of HIV/STI risk among AMGRM. Venue-specific interventions that strengthen peer networks, improve condom negotiation capacity, and integrate digital health strategies are needed to reduce risk and enhance engagement in HIV prevention and care.

## Introduction

Amidst ongoing global migration flows, Italy continues to serve as a critical entry point for African asylum seekers and refugees seeking protection and economic opportunity ^1–6^. Within this precarious landscape, many are driven into informal economies such as sex work to survive restrictive immigration policies, economic exclusion, and racialized labor markets^7–10^. Among these populations, African male and gender-diverse refugee/migrant workers (AMGRMWs), especially those identifying as transgender or gender non-conforming, confrontcompounded forms of marginalization shaped by racism, gender-based violence, and legal precarity^11,12^. However, their strategies for navigating risk and sustaining livelihoods remain underexplored in public health and migration scholarship^13^.

With the increasing digitalization of sex economies, dating and hookup platforms such as Grindr, Planet Romeo, Telegram, and WhatsApp have emerged as vital tools for AMGRMWs to solicit clients, negotiate transactions, and manage safety. These platforms offer opportunities for increased discretion, client screening, and autonomy features particularly salient for undocumented migrants exposed to criminalization and public violence^14^. Yet, digital platforms are not inherently protective^15,16^. They often replicate structural inequities in access to health information, increase exposure to digital surveillance, and fail to address the embedded risks of online exploitation^17,18^.

In parallel, AMGRMWs frequently rely on in-person venues such as bars, parks, and public streets or on peer-based referral systems to source clients^14,19^. These alternative modalities, while rooted in community and trust, are also fraught with dangers, including physical violence, coercion, and lack of access to basic protections (Scorgie et al., 2013; Lyons et al., 2017). The venue through which AMGRMWs meet clients often reflects broader social hierarchies, resource access, and legal status, ultimately shaping health risks and service engagement^20,21^.

This study is among the first to integrate the Theory of Planned Behavior (TPB) with an intersectional framework to examine how client-seeking environments shape sexual health behaviors among African refugee and migrant sex workers in Europe. This study examines how AMGRMWs in Northern Italy use client-seeking, risk-management, and survival strategies, and how these everyday tactics shape sexual health practices and healthcare engagement. Rather than focusing on motivations for entering sex work, the study investigates the operational strategies participants use to identify clients, screen for risk, manage client power dynamics, and maintain health and safety within highly constrained environments. Using a mixed-methods approach integrating qualitative narratives with quantitative survey data, we analyze how different client-seeking venues influence safety practices, condom negotiation, STI disclosure, and healthcare engagement among AMGRMWs in Verona, Turin, and Milan, drawing on the TPB to interpret how attitudes, perceived social norms, and perceived behavioral control shape sexual-health decision-making in these contexts.

The TPB posits that behavior is driven by behavioral intentions, which in turn are shaped by three core constructs: attitudes toward the behavior, subjective norms, and perceived behavioral control. In the context of refugee male sex work, attitudes may include beliefs about the benefits and risks of condom use or disclosing STI status; subjective norms reflect perceived expectations from peers, clients, and community organizations around protection and disclosure; and perceived behavioral control captures sex workers’ sense of agency in negotiating condom use, refusing unsafe clients, or accessing health services across different client-seeking venues. By situating client-seeking strategies and safety practices within a TPB framework, this study examines how structurally constrained environments interact with individual intentions to shape sexual-health behaviors among AMGRMWs in Northern Italy.

## Method

### Study Design

This study employed a sequential exploratory mixed-methods design^22^ to investigate how AMGRMWs in Northern Italy navigate client solicitation, safety strategies, and health access through various client engagement platforms. The design integrated in-depth qualitative inquiry with a structured quantitative survey to capture the lived complexity and population-level patterns shaping AMGRMWs experiences. The design and interpretation of both strands were informed by Intersectionality Theory, which provided a conceptual lens for examining how multiple social identities and structural conditions jointly shape participants’ experiences, and by the Theory of Planned Behavior (TPB)^23^, which guided our focus on how attitudes, perceived social norms, and perceived behavioral control influence client-seeking strategies, safety practices, and sexual-health behaviors.

The quantitative sample size (n = 150) was determined based on feasibility and the need to ensure adequate representation across key client-seeking venues, while maintaining sufficient cell counts for categorical analyses. Given the exploratory nature of the study and the use of Fisher’s Exact Test for small subgroup comparisons, this sample size was considered appropriate to detect meaningful associations between client-seeking environments and sexual health outcomes.

### Study Setting and Participants

Data collection was conducted in Verona and Turin, Italy, in collaboration with *Circolo Pink (Pink Refugees)*, a community-based organization that provides support services and safe spaces for AMGRMWs. This partnership facilitated participant recruitment and ensured a trusted, culturally sensitive environment for data collection.

### Measures

Table 1 summarizes the socio-demographic characteristics of male and gender-diverse refugee/migrant sex workers who participated in the survey. The mean age was 30.6 years (SD = 5.9), and participants reported initiating sex work at an average age of 23.3 years. Most had at least secondary education, with nearly three-quarters completing senior secondary/vocational training or tertiary education, indicating relatively high educational attainment despite current socioeconomic precarity. The vast majority identified as male, with a small proportion identifying as transgender or non-binary. Over half were single and about one-third were married, and approximately half reported having no children, while the remainder had one or more children.

Religious affiliation and religiosity were salient features of the sample. Most participants reported a religious affiliation most commonly Christianity, followed by Islam and African Traditional Religion with only a minority indicating no religion. Self-rated religiosity ranged from extremely religious to not religious, with many positioning themselves as moderately or “maybe” religious. Almost nine in ten participants held refugee status, with smaller proportions on other forms of protection or uncertain about their legal status, underscoring the precarious migration context. Length of stay in Italy varied, though the majority had lived in the country for at least one year and more than one-third for over two years, suggesting a mix of recent arrivals and more established migrants.

Countries of origin clustered in West and Central Africa, with Nigeria comprising over half of the sample, and additional representation from Ghana, Cameroon, Côte d’Ivoire, Gambia, Liberia, and Senegal. Participants predominantly identified as gay, with the remainder identifying as bisexual. Sexual role was relatively evenly distributed between those identifying as top and versatile, with a smaller proportion identifying as bottom. Most respondents reported no employment outside of sex work and modest monthly earnings, and the majority saw between one and five clients per day, with a minority reporting six to ten daily clients. Together, these characteristics portray a relatively young, religiously engaged, and socioeconomically constrained group of male and gender-diverse refugee/migrant sex workers with diverse family responsibilities and migration trajectories in the Italian context.

### Qualitative Data Collection

Between December 2023 and January 2024, twenty in-depth interviews (IDIs) and two focus group discussions (FGDs) were conducted with AMGRMWs. Interviews were led by bilingual researchers and supported by trained peer research assistants. Interview sessions took place in confidential, community-trusted locations, with participants choosing their preferred interview language (English or Italian). Informed consent was obtained before participation. The semi-structured interview guide included open-ended questions such as: i. “Where do you often meet your clients?” ii. “Which apps or online platforms do you use?” iii. “How do you negotiate with clients online?”, iv. “Have you ever faced problems or risks after meeting clients through apps?” v. “How did you first learn about these apps or platforms?”.

Additional probes explored participants’ experiences of online deception, physical violence, threats, safety strategies, mental health impacts, and barriers to reporting or seeking help. Participants were encouraged to share both positive and negative experiences, including strategies they used to enhance safety, avoid risks, and navigate client interactions through digital platforms.

Interviews and FGDs were audio-recorded with consent, transcribed verbatim, and translated into English where necessary. Translations prioritized accuracy and preservation of emotional tone and context.

Data collection continued until thematic saturation was achieved, defined as the point at which no new themes or substantive insights emerged from additional interviews. Saturation was reached after approximately 18 interviews, with two additional interviews conducted to confirm thematic completeness.

Eligibility criteria included being a Sub-Saharan African refugee or asylum seeker, 18 years or older, residing in Italy, self-identifying as a sex worker, and having engaged in transactional sex within the previous six months. Participants needed fluency in either English or Italian. Recruitment involved peer referrals, snowball sampling, and venue-based outreach by community collaborators with established trust networks.

### Quantitative Data Collection

Insights from the qualitative phase informed the development of a structured survey administered to 150 AMGRMWs. The survey measured, i. Frequency and type of app/platform use, ii. Methods of online negotiation and client arrangement, iii. Experiences of risk or harm, including online deception, physical violence, emotional abuse, and threats, iv. Safety strategies and help-seeking behaviors, v. Influence of peer and client networks on platform adoption, vi. Sociodemographic characteristics relevant to digital sex work practices. Surveys were distributed via REDCap, a secure, mobile-compatible platform to ensure anonymity and accessibility.

### Funding

This study was supported by the National Institute of Mental Health under award number P30MH062294, with funds administered by the Center for Interdisciplinary Research on AIDS at the Yale School of Public Health. The content is solely the responsibility of the authors and does not necessarily represent the official views of the Center for Interdisciplinary Research on AIDS at Yale, the National Institute of Mental Health, or the National Institutes of Health.

### Ethical Considerations

The study received ethical approval from the University of Rochester Research Subjects Review Board (STUDY00007858), The National Ethics Committee for Clinical Trials of Public Research Bodies, and the Istituto Superiore di Sanit‡ in Italy (AOO-ISS - 04/07/2023 - 0031228). Participants were informed about the study’s objectives, procedures, potential risks, and benefits. Written informed consent was obtained from participants. For those with limited literacy, the consent form was read aloud. Data confidentiality was strictly upheld, with all identifying information anonymized in data handling and reporting.

### Qualitative Data Analysis

Qualitative data were analyzed using summative content analysis. Two researchers independently conducted open coding of transcripts, identifying emergent patterns around key areas: digital platform use, transitions into sex work, negotiation practices, safety strategies, exposure to risks (including violence, threats, and emotional harm), and the role of peer support networks. Codes were iteratively refined into major thematic categories, including newly elaborated subthemes related to psychological impacts and barriers to reporting violence. Discrepancies were resolved through discussion, and final themes were developed to reflect both structural and psychosocial dimensions of participants’ experiences.

### Quantitative Data Analysis

We employed a combination of descriptive statistics and inferential tests to examine participants’ demographic, socioeconomic, and behavioral characteristics in relation to their reported venues for meeting clients. Descriptive statistics were used to summarize key participant characteristics, including age, income level, legal status, educational attainment, and awareness of HIV self-testing kits. Frequencies and percentages were reported for categorical variables to provide a clear overview of the sample distribution.

To evaluate associations between categorical variables, such as meeting venues and health-related behaviors (e.g., condom use, awareness of STI status, and motivations for entering sex work), we utilized Fisher’s Exact Test. This method was chosen over the Chi-square test due to small sample sizes in several subgroups, particularly those involving online client engagement, brothel-based work, or referrals. Fisher’s Exact Test provides accurate p-values without relying on assumptions about expected cell frequencies, making it appropriate for analyzing contingency tables with sparse data.

Additionally, we used two-proportion z-tests to assess whether the proportion of participants with a specific characteristic (e.g., awareness of HIV self-testing kits) differed significantly between two groups, those who used online platforms to meet clients and those who did not. This test allowed us to identify statistically meaningful differences in proportions and better understand potential disparities in knowledge or behaviors between subgroups. A significance threshold of p < 0.05 was used for all inferential analyses. All the analyses were conducted in RStudio-2025.05.0-496.

Missing data were assessed for all key variables prior to analysis. Analyses were conducted using available-case (complete-case) approaches, whereby participants with missing responses for specific variables were excluded from the corresponding analyses. The extent of missing data was minimal and primarily due to non-response on sensitive behavioral questions (e.g., condom use), resulting in reduced denominators for certain outcomes. No imputation procedures were applied due to the exploratory nature of the study and the small size of some subgroups.

## Results

### Qualitative Results

The qualitative findings reveal how AMGRMWs in Northern Italy actively navigate client-seeking, safety management, and survival within a complex and often precarious social environment, and how these practices reflect attitudes toward risk, perceived social expectations, and perceived control over client interactions consistent with the Theory of Planned Behavior. Participants described how everyday work practices are shaped by the need to balance income generation with personal safety, discretion, and risk reduction. Their narratives demonstrate that locating clients is not simply an economic activity, but a process embedded within broader strategies for avoiding violence, minimizing exposure to law enforcement, and managing uncertainty in client interactions.

Participants reported relying on multiple client-seeking environments, including digital platforms, public solicitation in urban spaces, and peer-based referral systems. These environments were not used in isolation. Instead, sex workers frequently moved between them depending on perceived safety, access to technology, housing conditions, and the availability of trusted networks. Across accounts, participants emphasized that each client-seeking strategy carries distinct benefits and risks, requiring sex workers to continuously adjust their practices to maintain control over their working conditions.

Three primary themes emerged from the qualitative analysis: **(1) client-seeking strategies in the Italian sex economy, (2) risk management and safety tactics, and (3) navigating client power and exploitation**. Together, these themes illustrate how sex workers develop practical survival strategies to manage risk within the sex economy.

#### 1. Client-Seeking Strategies in the Italian Sex Economy

Participants described several approaches for locating clients within the Italian sex economy. These approaches included digital platforms, in-person solicitation in public venues, and peer-based referral systems. Sex workers often moved between these strategies depending on safety concerns, technological access, and social networks. Their narratives illustrate how client-seeking strategies are embedded within broader considerations of discretion, security, and mobility.

##### 1.1 Digital Platforms as Primary Client-Seeking Tools

For many participants, digital platforms represented the primary avenue for locating and communicating with clients. Applications such as Grindr, Planet Romeo, Telegram, and WhatsApp were frequently mentioned as tools that allow sex workers to interact with clients before arranging in-person meetings. Participants emphasized that these platforms provided greater discretion and control compared with street-based work, allowing them to communicate with potential clients while remaining in private spaces.

One participant explained how digital platforms provide the ability to select clients more carefully:

“Mostly I find clients online. I use apps like Grindr or Planet Romeo. It’s safer than standing on the street, and I get to choose who I want to meet.” (Participant 3)

Participants also described how digital platforms blur the boundaries between social and commercial interactions. In many cases, the same applications are used for dating, social networking, and client-seeking, making it difficult to distinguish clearly between personal and transactional relationships.

“I use the app for both clients and sometimes for dates. Some people on there know what they want, others don’t. If they’re respectful and I feel safe, I might meet them.” (Participant 19)

Messaging platforms such as Telegram and WhatsApp were also described as important tools for maintaining smaller and more controlled client networks. Participants explained that these platforms allowed them to interact within more private groups or communicate directly with individuals who had already been introduced through trusted networks.

“I’ve also met clients through Telegram groups. There are private chats where people post ads or ask for company.” (Participant 4)

Another participant described how WhatsApp enables a more selective client base:

“Now I use WhatsApp. I don’t advertise openly, but I have a small network. If someone is interested, they contact me. It’s safer, and I can choose who to meet.” (Participant 7)

##### 1.2 In-Person Solicitation in Public and Nightlife Venues

Despite the widespread use of digital platforms, several participants described continuing to meet clients in physical spaces such as train stations, parks, bars, and clubs. These environments were described as recognizable locations within the local sex economy where potential clients and sex workers can identify one another.

One participant described how public areas operate as informal marketplaces for sexual services:

“You can go to Termini, especially at night. You walk around, and people know what you’re there for. They come up and ask you directly how much or what services you offer.” (Participant 10)

In such settings, client interactions occur through direct conversation, allowing sex workers and clients to negotiate services immediately. However, participants also emphasized that public solicitation carries significant risks, particularly in relation to law enforcement and public harassment.

Several sex workers explained that police presence in these areas has influenced their decision to move toward digital platforms. One participant described how repeated police encounters forced him to change his approach to client-seeking:

“In the beginning, I used to go to the train station. But the police came too often, so now I prefer using my phone.” (Participant 6)

Bars and nightclubs were also identified as locations where client interactions could occur more informally through social engagement. In these spaces, sex workers may interact with potential clients through dancing, drinking, or conversation.

“There are certain bars where people know sex workers go. You go, dance, drink, and people come up to you. It’s not always safe, but it works.” (Participant 13)

Although nightlife venues provide opportunities for meeting clients, participants acknowledged that these environments are unpredictable and may expose sex workers to discrimination, intoxicated clients, or other forms of risk.

For some participants, however, public venues remain one of the few available options for locating clients. Sex workers who lack access to digital platforms or established networks may rely on street-based solicitation despite the associated risks.

“I just go out and stand somewhere, or sometimes I go to a gay club. There’s also a park where people know trans girls go to find clients. It’s not very safe, but we don’t have many other options.” (Participant 1)

##### 1.3 Peer Referral Networks

Peer referral systems also emerged as an important strategy for locating clients. Participants described relying on friends and colleagues within the sex work community to introduce them to potential clients. These networks function as systems of mutual support where sex workers share information about safe locations, pricing strategies, and reliable clients.

One participant described how peer referrals operate as collective safety mechanisms:

“Sometimes friends introduce me to clients. We help each other out and look out for one another.” (Participant 5)

Participants also described how satisfied clients may refer them to other potential clients, allowing sex workers to build networks through word-of-mouth recommendations.

“Once you have one or two regulars, they introduce you to their friends. That’s how the business grows, especially if you treat them well.” (Participant 12)

In some cases, knowledge about client-seeking practices was shared informally among migrants living in the same housing environments. Sex workers described learning about the sex economy through conversations with others who had already begun working.

“Even in the camp or the hostel, you meet other guys who are doing sex work. They tell you where to go, what to say, and how much to charge. That’s how I learned, too.” (Participant 14)

#### 2. Risk Management and Safety Tactics

Participants described several strategies used to reduce the risks associated with client interactions. These strategies included screening clients prior to meetings and relying on peer monitoring systems for protection.

##### 2.1 Client Screening Before Meetings

A common safety strategy involved communicating with clients prior to meeting them in person. Participants described using conversation and profile information to assess whether potential clients appeared trustworthy.One participant explained how he evaluates potential clients before agreeing to meet:

“When someone writes to me, I talk to them a bit first. If I feel something is off, I don’t meet them.” (Participant 17)

Digital platforms also allow sex workers to avoid anonymous or suspicious profiles by blocking individuals who appear unsafe.

“With the app, you can see who they are. I don’t accept anonymous profiles. If they don’t show their face or don’t want to talk first, I block them.” (Participant 3)

##### 2.2 Peer Safety Monitoring

Participants also described relying on friends and colleagues to monitor their safety during client meetings. These informal systems allow sex workers to remain connected with trusted individuals who can respond if something goes wrong. One participant described how he shares his location with a friend when meeting clients:

“When I go out to meet someone, I always tell my friend. We share location on the phone. If I don’t respond, she knows something is wrong.” (Participant 5)

#### 3. Navigating Client Power and Exploitation

Participants described several forms of exploitation and aggression within client encounters. These experiences illustrate the unequal power dynamics that often characterize interactions between sex workers and clients.

##### 3.1 Client Violence and Theft

Several participants described incidents of physical violence or robbery during client encounters. These incidents often occurred when sex workers met clients in private locations where assistance was not immediately available. One participant described being attacked by a client who initially appeared legitimate:

“One time, a man came to my place pretending to be a client, but he attacked me. He beat me and took my phone and money. I was alone, I couldn’t do anything.” (Participant 2)

Other participants described situations in which clients refused to pay after sexual encounters.

“Some clients seem nice, but when they get what they want, they become aggressive or don’t want to pay.” (Participant 16)

##### 3.2 Discrimination and Gender-Based Harassment

Participants who identified as transgender described experiencing harassment and hostility from some clients. These encounters often involved verbal abuse and threats of violence. One participant described how clients sometimes reacted upon discovering her gender identity:

“Some men come and when they see I’m trans, they start insulting me or even try to hit me. It happens a lot. People don’t respect us.” (Participant 11)

### Quantitative Results

The quantitative component examined how client-seeking environments shape safety practices, sexual health behaviors, and healthcare engagement among AMGRMWs in Northern Italy. Consistent with the qualitative findings, the quantitative analysis focuses on operational strategies participants use to locate clients, negotiate sexual health risks during encounters, and navigate healthcare systems while working within contexts of legal and social precarity.

Descriptive statistics were first used to examine the distribution of client-seeking venues among participants. Inferential analyses were then conducted using Fisher’s Exact Test to evaluate associations between meeting venues and key behavioral outcomes, including knowledge of clients’ sexual health status, condom use, STI disclosure practices, engagement in unprotected sex under client pressure, and immigration-related barriers to healthcare.

### Distribution of Client-Seeking Venues

The analysis revealed that referral-based networks were the most common strategy used by participants to locate clients. Specifically, 94 out of 150 participants (62.7%) reported primarily meeting clients through referrals from friends or colleagues within sex work networks. Bars, pubs, or clubs were the second most frequently reported venue, used by 21 participants (14.0%), followed by street-based solicitation reported by 20 participants (13.3%). A smaller proportion of participants reported primarily meeting clients through online platforms such as Grindr or Planet Romeo (n = 10, 6.7%), while only five participants (3.3%) reported primarily working in brothel or massage-parlor environments.

These findings suggest that peer-based referral systems represent the dominant structure through which AMGRMWs access clients within the Italian sex economy. The reliance on referrals likely reflects the role of community networks in facilitating safer interactions and providing access to trusted clients, highlighting how subjective norms within peer networks shape intentions to seek clients through venues perceived as safer and more controllable.

### Client-Seeking Venues and Knowledge of Clients’ Sexual Health Status.

Participants were asked whether they were aware of their clients’ sexual history or STI status before engaging in sexual activity. Overall, 51 participants (34.0%) reported knowing their partner’s sexual health status, while 99 participants (66.0%) reported that they did not have this information before sexual encounters.

The analysis revealed a statistically significant relationship between client-meeting venue and awareness of partners’ STI status (Fisher’s Exact Test = 15.78, p = 0.003, Cramér’s V = 0.32). Sex workers operating in brothel environments reported the highest level of awareness, with all five participants in this subgroup (100%) reporting knowledge of their clients’ STI status. Sex workers meeting clients in nightlife venues also reported moderate levels of awareness, with 9 out of 21 participants (42.9%) indicating they knew their partner’s sexual health status prior to sexual activity.

Among sex workers relying on referral networks, 31 of 94 participants (33.0%) reported awareness of clients’ sexual health histories, while the remaining 63 participants (67.0%) did not have this information. Street-based sex workers reported lower levels of awareness, with only 6 out of 20 participants (30.0%) reporting knowledge of their clients’ STI status. Notably, none of the participants who met clients online reported knowing their partners’ sexual health histories before engaging in sexual activity (0 out of 10 participants).

These findings suggest that environments characterized by repeated interactions or structured settings may facilitate greater communication about sexual health compared with environments characterized by anonymity or rapid encounters.

### Condom Use Across Client-Seeking Venues

Participants were also asked whether they used condoms during anal sex with clients. Due to missing responses on sensitive behavioral items, the analytic sample for condom use was reduced to n = 137. Among the participants who responded to this question, 112 out of 137 participants (81.8%) reported using condoms during sexual encounters, while 25 participants (18.2%) reported not using condoms.

The results revealed a statistically significant association between meeting venue and condom use (Fisher’s Exact Test = 13.57, p = 0.009, Cramér’s V = 0.31). Sex workers operating in brothel settings reported universal condom use, with all four participants in this subgroup (100%) reporting condom use. Street-based sex workers also reported high levels of condom use, with 17 out of 18 participants (94.4%) indicating that they used condoms during sexual encounters.

Participants meeting clients in bars or nightlife venues reported slightly lower levels of condom use, with 18 out of 21 participants (85.7%) reporting condom use and 3 participants (14.3%) reporting that condoms were not used. Among sex workers relying on referral networks, 70 out of 86 participants (81.4%) reported condom use, while 16 participants (18.6%) reported not using condoms.

In contrast, sex workers meeting clients online reported the lowest levels of condom use. Only 3 out of 8 participants (37.5%) in this subgroup reported using condoms, while 5 participants (62.5%) reported not using condoms during sexual encounters.

These findings suggest that digitally mediated encounters may present challenges for condom negotiation compared with interactions occurring through more structured or community-based environments.

### STI Disclosure Practices

Participants were asked whether they would notify their clients if they had an STI. Overall, 59 out of 150 participants (39.3%) reported that they would notify their clients about STI infection, while 91 participants (60.7%) indicated that they would not disclose this information.

The analysis revealed a statistically significant association between meeting venue and willingness to disclose STI infection (Fisher’s Exact Test = 14.68, p = 0.005, Cramér’s V = 0.31). Street-based sex workers reported the highest willingness to notify clients, with 14 out of 20 participants (70.0%) indicating they would disclose STI infection.

Among sex workers meeting clients in bars or nightlife venues, 11 out of 21 participants (52.4%) reported that they would notify clients about STI infection. Sex workers operating in brothel settings also reported relatively high disclosure levels, with 3 out of 5 participants (60.0%) indicating that they would notify clients.

In contrast, sex workers relying on referral networks reported lower levels of STI disclosure, with only 29 out of 94 participants (30.9%) indicating willingness to notify clients. Online-based sex workers reported the lowest disclosure levels, with only 2 out of 10 participants (20.0%) indicating they would inform clients about STI infection.

### Unprotected Sex Under Client Pressure

Participants were also asked whether they had ever engaged in sexual activity without protection after being offered additional money or other incentives. Overall, 78 participants (52.0%) reported that they had accepted unprotected sex due to financial or situational incentives, while 72 participants (48.0%) reported that they had not.

The analysis revealed a statistically significant association between meeting venue and acceptance of unprotected sex (Fisher’s Exact Test = 12.94, p = 0.011, Cramér’s V = 0.29). Street-based sex workers reported the highest prevalence of accepting unprotected sex, with 14 out of 20 participants (70.0%) indicating that they had engaged in unprotected sex under client pressure.

Participants working in brothel settings reported the highest proportion overall, with all five participants (100%) reporting acceptance of unprotected sex due to incentives, although this subgroup was small. Sex workers relying on referral networks also reported relatively high levels of accepting unprotected sex, with 49 out of 94 participants (52.1%) indicating that they had accepted unprotected sex.

By contrast, sex workers meeting clients online reported the lowest likelihood of accepting unprotected sex due to financial incentives, with only 2 out of 10 participants (20.0%) reporting such experiences.

### Integrated Mixed-Methods Findings

To better interpret these quantitative patterns, the results were integrated with qualitative findings using a mixed-methods joint display. The integrated analysis illustrates how operational survival strategies described in interviews help explain the statistical relationships observed in the survey data.

For example, the significant association between meeting venue and awareness of clients’ STI status (p = 0.003) can be understood through participants’ descriptions of referral networks where sex workers share information about clients and help one another screen potentially unsafe encounters. Similarly, the lower levels of condom use observed among online-based sex workers (p = 0.009) correspond with qualitative accounts describing how negotiations conducted through digital platforms may reduce sex workers’ ability to renegotiate condom use once meetings occur.

Differences in STI disclosure (p = 0.005) and acceptance of unprotected sex due to financial incentives (p = 0.011) also align with interview narratives describing how the structure of client interactions influences sex workers’ bargaining power during negotiations. Finally, the higher prevalence of immigration-related barriers to healthcare among street-based sex workers (p = 0.025) reflects participants’ accounts of policing, visibility, and fear of institutional discrimination.

### Reflexivity and Researcher Positionality.

This study was conducted by a multidisciplinary research team with expertise in global health, migration studies, qualitative and quantitative methods, and community-based participatory research. Members of the team brought diverse professional and personal perspectives, including backgrounds in behavioral health, public health sciences, sociology, and data analytics. This diversity enriched both the design and interpretation of findings but also required deliberate attention to positionality.

Several members of the research team identify as immigrants or members of historically marginalized communities, which facilitated sensitivity to issues of stigma, racialization, and precarity. At the same time, others held positions of institutional privilege within U.S. and European universities. We acknowledged that these positionalities could influence the questions we asked, how participants’ narratives were interpreted, and the weight assigned to findings. To address this, coding and analysis were conducted collaboratively by pairs of researchers with different methodological backgrounds, and discrepancies were discussed openly until consensus was reached.

Our collaboration with Circolo Pink (Pink Refugees), a community-based organization in Verona, was essential in ensuring cultural relevance and participant trust. Peer research assistants, themselves members of the refugee sex worker community, contributed to recruitment, interpretation of transcripts, and contextualization of findings. Their involvement helped mitigate risks of outsider bias and ensured that participant voices were authentically represented.

We also recognized power dynamics between researchers and participants, particularly around language, legal status, and gender identity. To minimize these imbalances, interviews were conducted in the preferred language of participants, and data collection sites were chosen collaboratively to maximize safety and comfort. Throughout the study, the team engaged in reflexive discussions about how our identities, assumptions, and institutional affiliations might shape the knowledge being produced. This reflexive practice strengthened the credibility, transparency, and trustworthiness of the mixed methods findings.

## Discussion

This study advances understanding of how AMGRMWs in Northern Italy navigate client-seeking, safety management, and sexual health within conditions shaped by migration precarity, racialization, stigma, and exclusion from formal labor markets. Viewed through the TPB, the venue through which participants meet clients is not merely a logistical detail; it is a social and structural context that shapes attitudes toward risk, perceived social norms within peer and client networks, and perceived behavioral control over condom negotiation, STI disclosure, and healthcare engagement. This study is an important contribution because much of the literature on sex work and HIV still privileges individual behavior over the interaction between work environment, legal vulnerability, and social inequality^24–26^.

A central finding is that client-seeking environments create distinct configurations of attitudes, norms, and control rather than fitting neatly into “safe” and “unsafe” categories. Referral-based networks were the dominant mode of client acquisition, indicating that peer-mediated pathways are foundational within this local sex economy and strongly shape subjective norms. This pattern suggests that social capital and trust are critical survival resources for refugee sex workers in Italy^27,28^, especially where formal institutional protection is weak; prior work similarly describes peer networks as informal harm-reduction systems through which sex workers share information about pricing, safety, and client reliability^29–31^. In our data, that normative role was reflected in referral-based sex workers’ relatively strong condom use and moderate awareness of clients’ STI status, indicating attitudes that value protection and a sense of control in navigating known clients, while low willingness to disclose one’s own STI status and substantial acceptance of unprotected sex under financial pressure point to norms and economic constraints that temper intentions toward safer behavior. Peer support can thus bolster protective intentions but cannot fully offset structural deprivation that limits perceived behavioral control over refusing risky propositions ^29–31^.

The findings on digital client-seeking are especially important from a TPB perspective. Qualitative accounts presented apps and messaging platforms such as Grindr, Planet Romeo, Telegram, and WhatsApp as tools of discretion and preliminary client screening that appear to enhance perceived control in avoiding police and public harassment. Digital technologies allowed sex workers to select clients, communicate from private spaces, and block suspicious profiles, shaping attitudes that online encounters are more manageable and less visibly risky. However, quantitative findings complicate any assumption that online client-seeking is inherently protective. Participants who met clients online had the lowest awareness of clients’ STI status, the lowest condom use, and the lowest willingness to disclose STI infection, alongside lower awareness of HIV self-testing. Interpreted via TPB, these patterns suggest that although online contexts may foster positive attitudes about privacy and autonomy, anonymity and weaker peer oversight undermine subjective norms that support disclosure and consistent condom use, and reduce perceived control once in-person encounters occur. Digital access therefore does not automatically translate into health-protective intentions or behaviors but instead reconfigures the balance between discretion, accountability, and negotiability^32^.

This divergence between digital access and health protection is conceptually important. Online sex workers may appear more socially mobile or technologically connected, yet remain excluded from prevention messaging, peer-led education, and trusted healthcare pathways that shape norms and behavioral control^33,34^. Recent scholarship on digital health inequities among migrant and marginalized populations similarly cautions against equating digital connectivity with digital health literacy or service access^34^. In this study, that disconnect helps explain why the apparent autonomy of online solicitation coexisted with poorer sexual-health indicators and weaker intentions to notify clients about STI infection. TPB-informed HIV prevention strategies for digitally mediated sex work will therefore need to specifically target attitudes about risk in anonymous encounters, leverage influential peers within existing app-based networks to shift norms, and enhance perceived control by building negotiation skills and clear pathways to confidential testing and care within digital ecosystems.

Street-based work revealed a different but equally complex TPB profile. Street-based participants described police presence, visibility, harassment, and institutional fear as part of everyday work, conditions that likely depress perceived behavioral control over where and how they can solicit clients or seek care. These findings resonate with evidence that punitive legal environments, immigration insecurity, and policing intensify sex workers’ vulnerability and undermine access to services^35,36^. Yet street-based participants also reported relatively high condom use and the highest willingness to disclose STI infection, suggesting that repeated interactions with local clients and immediate face-to-face negotiation may foster attitudes that prioritize visible protective practices and norms of disclosure within certain micro-environments. At the same time, street-based sex workers faced substantial immigration-related healthcare barriers and high acceptance of unprotected sex under client pressure, illustrating how structural vulnerability and economic dependence constrain the translation of protective intentions into consistent behavior^37^. From a TPB lens, this underscores that strong intentions toward safer practices can coexist with low perceived control when financial and legal pressures are acute.

The small brothel or massage-parlor subgroup provides an additional contrast. These participants reported universal awareness of clients’ STI status and universal condom use, indicating strong pro-protection norms within more structured environments and higher perceived control over enforcing venue rules. Yet they also universally reported accepting unprotected sex due to incentives, suggesting that intense client pressure and economic dependence can override protective attitudes and norms under specific circumstances. Even with small cell sizes, this pattern highlights the limits of inferring safety from single TPB constructs such as attitudes or norms without considering the powerful influence of financial incentives on perceived control and actual behavior. The finding aligns with literature showing that indoor environments can offer protection in some domains while concentrating exploitation and bargaining asymmetries in others^38,39^.

Qualitative accounts of violence, theft, refusal of payment, and gender-based harassment deepen TPB interpretation. Participants were not only trying to prevent HIV or STIs; they were simultaneously managing threat of robbery, assault, humiliation, and income loss, all of which shape attitudes toward condom negotiation and disclosure as potentially dangerous or costly. Experiences of violence and discrimination also influence subjective norms, for example when transgender participants learn that disclosure of gender identity or STI status can trigger abuse, reinforcing norms of concealment. These narratives illustrate that “safety management” in sex work is multidimensional and that the determinants of behavioral intentions span bodily security, economic survival, and protection from stigma and policing, consistent with intersectional and rights-based frameworks^39–41^.

The intersectional framing is therefore one of this study’s major strengths and complements TPB by situating attitudes, norms, and perceived control within broader structures. Although most participants identified as men, transgender and non-binary participants described additional layers of harassment and hostility linked to gender identity, which likely depress perceived control and foster norms of secrecy around both identity and health status. Existing evidence from Italy documents that migrant transgender sex workers face intersecting barriers related to transphobia, xenophobia, language exclusion, and insecure legal status^27,28,42,43^. The present findings fit within that pattern while adding a specific focus on AMGRMWs, a population underrepresented in European HIV and migration research^28,42^, and demonstrate that TPB constructs are themselves shaped by migration control, racialization, gender policing, and economic marginality.

The healthcare implications in this study are substantial when viewed through TPB. Venue type was associated with not only condom use and STI disclosure but also awareness of clients’ sexual-health status, HIV self-testing awareness, and immigration-related barriers to care, all of which reflect underlying attitudes, norms, and perceived control regarding testing and engagement with services. The particularly low awareness of HIV self-testing among online sex workers suggests missed opportunities to cultivate favorable attitudes toward self-testing, establish norms of regular screening within digital peer networks, and increase perceived control by emphasizing privacy and convenience. Complementing prior work among AMGRMWs in Italy^44^, our findings indicate that TPB-informed interventions could embed self-testing promotion within trusted digital channels and peer networks, framing testing as both normative and feasible despite legal and social precarity.

Policy implications also emerge clearly from a TPB perspective. First, HIV and STI interventions for refugee and migrant sex workers in Italy should be venue-sensitive, recognizing that different environments foster different attitudes toward risk, social expectations around disclosure and condom use, and levels of perceived control^44^. Second, peer-led models should be strengthened because referral systems already function as normative infrastructures that can be leveraged to promote harm-reduction norms and empower sex workers to collectively resist unsafe client demands^31^. Third, digital health interventions should be designed to shift norms and enhance perceived control within the specific platforms sex workers use, rather than assuming generic online outreach will influence intentions or behaviors^33^. Finally, structural reforms that reduce legal precarity, punitive enforcement, and healthcare exclusion are essential^27,42,45^, because TPB constructs themselves are shaped and constrained by law, policy, and material conditions; interventions that ignore these structural determinants will struggle to translate changed attitudes into sustainable behavior change^46^.

Methodologically, the mixed-methods design allowed us to map TPB constructs across qualitative narratives and quantitative patterns. The survey identified statistically significant associations between venue and outcomes such as condom use, STI-status awareness, disclosure, and acceptance of unprotected sex, while qualitative data illuminated how attitudes, norms, and perceived control were formed and negotiated in practice. Importantly, the two strands did not simply converge but also revealed tensions for example, online work appearing to enhance perceived control over visibility but coinciding with weaker condom use and disclosure. These contradictions underscore that behavioral intentions are dynamic and context-dependent, reinforcing the value of integrating TPB with intersectional and structural analyses rather than treating it as an exclusively individual-level model.

Our study shows that client-seeking strategies among AMGRMWs in Northern Italy are best understood as adaptive survival practices shaped simultaneously by structural forces and TPB constructs. Different venues offer different combinations of discretion, income opportunity, trust, and danger, which in turn influence attitudes toward protection, perceived norms around safety and disclosure, and perceived control over negotiating with clients or accessing care. Public health responses that focus only on individual risk behavior without addressing the environments in which attitudes, norms, and control are formed will remain insufficient. More effective interventions will integrate peer support, culturally responsive sexual-health services, digital outreach, and structural reforms that expand the actual and perceived capacity of refugee sex workers to act on their intentions for safety and health.

## Conclusion

Our study shows that client-seeking among AMGRMWs in Northern Italy is a survival strategy shaped by immigration precarity, racialized labor exclusion, stigma, and constrained access to formal employment and health services. Using the Theory of Planned Behavior, we demonstrate that different client-seeking venues referral networks, digital platforms, streets, nightlife venues, and brothels, foster distinct constellations of attitudes toward risk, subjective norms around protection and disclosure, and perceived behavioral control over negotiating with clients and engaging in care. Digital platforms enhanced discretion and reduced visibility to policing but were associated with the lowest levels of condom use, STI-status awareness, disclosure, and HIV self-testing awareness, underscoring that connectivity alone does not guarantee protective intentions or behaviors. Peer referral systems functioned as key normative infrastructures that facilitated trust, information sharing, and relatively high condom use, yet economic pressures and structural vulnerability still pushed many sex workers to accept unprotected sex or withhold STI disclosure. A rights-based, TPB-informed response must therefore combine structural reforms (reducing legal and healthcare exclusion) with venue-specific, peer-led, and digitally adapted interventions that strengthen protective attitudes, reshape norms, and expand the actual and perceived control of refugee sex workers over their safety, income, and health.

### Limitations

This study has several limitations that should be considered when interpreting the findings. First, the sample was recruited through community partners, peer referral, and venue-based outreach in specific cities in Northern Italy, which may limit generalizability to AMGRMWs in other Italian regions or European contexts with different legal, social, or service environments. Second, the cross-sectional quantitative design precludes causal inference; while we observed strong associations between client-seeking venues and sexual-health behaviors, we cannot determine the temporal direction of these relationships or rule out unmeasured confounding factors. Third, behavioral data such as condom use, STI disclosure, and acceptance of unprotected sex relied on self-report and may be affected by recall bias or social desirability, particularly given the stigmatized, criminalized, and precarious nature of sex work and migration status. Fourth, some venue categories, especially brothels and online-based work had relatively small subgroup sizes, which may have reduced statistical power and produced unstable estimates for certain outcomes. Fifth, while we applied the Theory of Planned Behavior to interpret attitudes, norms, and perceived control, we did not directly measure all TPB constructs with validated scales, and our use of proxy indicators may not capture the full complexity of participants’ decision-making processes. Finally, although qualitative methods enriched our understanding of structural and psychosocial contexts, participants who were most marginalized, fearful of institutions, or disconnected from community organizations may still be underrepresented, meaning that the most extreme forms of exploitation, violence, and health exclusion might not be fully reflected in these data.

## Figures and Tables

**Table 1.** Socio-Demographic Characteristics.

Variable	Category / Statistic	n (%) or Mean (SD)
**Age (years)**	Mean (SD)	30.6 (5.9)
	Range	23–34
**Age at starting sex work (years)**	Mean (SD)	23.3 (4.6)
**Monthly income (€)**	Mean (SD)	253.5 (212.4)
	Median (subsample)	~2,300
	Range (subsample)	1,000–6,000
**Education**	No formal education	7 (4.7)
	Primary or less	30 (20.0)
	Senior secondary/vocational	74 (49.0)
	Tertiary	39 (26.0)
**Gender identity**	Man	145 (96.7)
	Transgender	3 (2.0)
	Non-binary	2 (1.3)
**Marital status**	Single	86 (57.3)
	Married	53 (35.3)
	Divorced	10 (6.7)
	Widowed	1 (0.7)
**Number of children**	0	80 (53.3)
	1	25 (16.7)
	2	26 (17.3)
	3	13 (8.7)
	≥4	6 (4.0)
**Religious affiliation**	Christianity	71 (47.3)
	Islam	37 (24.7)
	African Traditional Religion	16 (10.7)
	Judaism	1 (0.7)
	No religion	25 (16.7)
**Rank of religiosity**	Extremely religious	8 (5.3)
	Very religious	15 (10.0)
	Moderately religious	25 (16.7)
	Maybe religious	32 (21.3)
	Not very religious	55 (36.7)
	Not religious	15 (10.0)
**Immigration status**	Refugee	131 (87.3)
	Subsidiary	6 (4.0)
	Motivo Speciale	8 (5.3)
	Don’t know	5 (3.3)
**Length of stay in Italy**	Less than 3 months	10 (6.7)
	3–6 months	21 (14.0)
	6 months–1 year	32 (21.3)
	1–2 years	30 (20.0)
	More than 2 years	57 (38.0)
**Country of origin**	Nigeria	86 (57.3)
	Ghana	16 (10.7)
	Cameroon	6 (4.0)
	Côte d’Ivoire	13 (8.6)
	Gambia	9 (6.0)
	Liberia	10 (6.7)
	Senegal	10 (6.7)
**Sexual orientation**	Gay	106 (70.7)
	Bisexual	44 (29.3)
**Sexual role**	Top	63 (42.0)
	Bottom	28 (18.7)
	Versatile	59 (39.3)
**Other job besides sex work**	Yes	32 (21.3)
	No	118 (78.7)
**Average daily clients**	1–5	133 (88.7)
	6–10	17 (11.3)

**Table 2.** Distribution of Client-Seeking Venues Among Participants

Client Meeting Venue	n	%
Through referrals	94	62.7
Bars / pubs / clubs	21	14.0
On the streets	20	13.3
Online platforms	10	6.7
Brothels / massage parlors	5	3.3
**Total**	**150**	**100**

**Table 3.** Client Meeting Venue and Awareness of Clients’ Sexual History or STI Status

Client Meeting Venue	Aware of Partner’s STI Status n (%)	Not Aware n (%)	Total
On the streets	6 (30.0)	14 (70.0)	20
Brothels / massage parlors	5 (100.0)	0 (0.0)	5
Bars / pubs / clubs	9 (42.9)	12 (57.1)	21
Through referrals	31 (33.0)	63 (67.0)	94
Online	0 (0.0)	10 (100.0)	10
**Total**	**51 (34.0)**	**99 (66.0)**	**150**

**Table 4.** Client Meeting Venue and Condom Use During Anal Sex

Client Meeting Venue	Condom Use n (%)	No Condom Use n (%)	Total
On the streets	17 (94.4)	1 (5.6)	18
Brothels / massage parlors	4 (100.0)	0 (0.0)	4
Bars / pubs / clubs	18 (85.7)	3 (14.3)	21
Through referrals	70 (81.4)	16 (18.6)	86
Online	3 (37.5)	5 (62.5)	8
**Total**	**112 (81.8)**	**25 (18.2)**	**137**

**Table 5.** Client Meeting Venue and Willingness to Notify Clients About STI Infection

Client Meeting Venue	Would Notify Client n (%)	Would Not Notify n (%)	Total
On the streets	14 (70.0)	6 (30.0)	20
Brothels / massage parlors	3 (60.0)	2 (40.0)	5
Bars / pubs / clubs	11 (52.4)	10 (47.6)	21
Through referrals	29 (30.9)	65 (69.1)	94
Online	2 (20.0)	8 (80.0)	10
**Total**	**59 (39.3)**	**91 (60.7)**	**150**

**Table 6.** Client Meeting Venue and Acceptance of Unprotected Sex Due to Incentives

Client Meeting Venue	Accepted Unprotected Sex n (%)	Did Not Accept n (%)	Total
On the streets	14 (70.0)	6 (30.0)	20
Brothels / massage parlors	5 (100.0)	0 (0.0)	5
Bars / pubs / clubs	8 (38.1)	13 (61.9)	21
Through referrals	49 (52.1)	45 (47.9)	94
Online	2 (20.0)	8 (80.0)	10
**Total**	**78 (52.0)**	**72 (48.0)**	**150**

**Table 8.** Mixed-Methods Joint Display Linking Quantitative and Qualitative Findings

Quantitative Finding	Statistical Result	Qualitative Explanation	Integrated Interpretation
Awareness of clients’ STI status varies by meeting venue	Fisher’s Exact Test = 15.78, p = 0.003, Cramér’s V = 0.32	Sex workers described referral networks where information about clients is shared among peers.	Peer networks function as informal harm-reduction systems.
Condom use differs across venues	Fisher’s Exact Test = 13.57, p = 0.009, Cramér’s V = 0.31	Participants reported difficulty renegotiating condom use during online-arranged encounters.	Digital client-seeking environments may reduce bargaining power during negotiations.
STI disclosure varies by venue	Fisher’s Exact Test = 14.68, p = 0.005, Cramér’s V = 0.31	Street-based sex workers described repeated interactions with local clients.	Communication norms differ across client environments.
Unprotected sex under incentives varies by venue	Fisher’s Exact Test = 12.94, p = 0.011, Cramér’s V = 0.29	Participants described stronger client pressure in visible working environments.	Structural conditions shape negotiation power.
Structural and immigration-related barriers to care by venue	Fisher’s Exact Test = 11.10, p = 0.025, Cramér’s V = 0.27	Street-based sex workers described fear of police and immigration authorities.	Structural marginalization shapes healthcare access.
